# Single Cell Spatial Transcriptomics of the Murine Embryonic Palate Links Pax9 to Patterning and Organization of Extracellular Matrix Components

**DOI:** 10.21203/rs.3.rs-5969552/v1

**Published:** 2025-02-19

**Authors:** Jeremie Oliver Piña, Resmi Raju, Evan Stipano, Aye Chan Myo, Ziyi Wang, Mitsuaki Ono, Parna Chattaraj, Masae Furukawa, Rena N. D’Souza

**Affiliations:** 1. Section on Craniofacial Genetic Disorders, *Eunice Kennedy Shriver* National Institute of Child Health and Human Development (NICHD), National Institutes of Health (NIH), Bethesda, MD, USA.; 2. Okayama University Graduate School of Medicine Dentistry and Pharmaceutical Sciences, Department of Molecular Biology and Biochemistry, Okayama, Japan.; 3. National Institute of Dental and Craniofacial Research (NIDCR), National Institutes of Health (NIH), Bethesda, MD, USA.

**Keywords:** Spatial Biology, Cleft Palate, Genomics, Single Cell, Gene Expression Profiling, Extracellular Matrix, Wnt, Transcriptome

## Abstract

Despite advances in understanding the morphological disruptions that lead to defects in palate formation, the precise perturbations within the signaling microenvironment of palatal clefts remain poorly understood. To explore in greater depth the genomic basis of palatal clefts, we designed and implemented the first single cell spatial RNA-sequencing study in a cleft palate model, utilizing the *Pax9*^−/−^ murine model at multiple developmental timepoints, which exhibits a consistent cleft palate defect. Visium HD, an emerging platform for true single-cell resolution spatially resolved transcriptomics, was employed using custom bins of 2×2 μm spatial gene expression data. Validation of spatial gene expression was then validated using custom designed Xenium In Situ mRNA spatial profiling and RNAscope Multiplex assays. Functional enrichment analysis revealed a palate cell-specific perturbation in Wnt signaling effector function in tandem with disrupted expression of extracellular matrix genes in developing mesenchyme. As a key step toward laying the framework for identifying key molecular targets these data can be used for translational studies aimed at developing effective therapies for human palatal clefts.

## Graphical Abstract

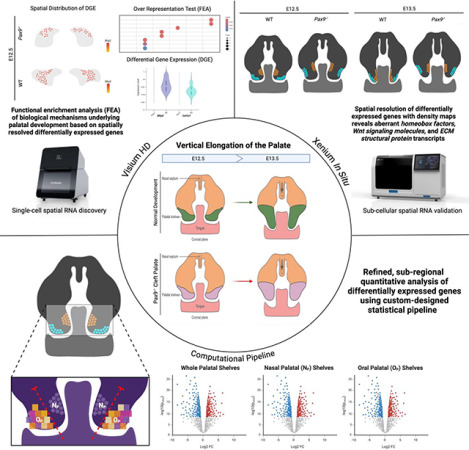

## INTRODUCTION

Home to some of the most intricate structures in the human body, the craniofacial complex requires tightly controlled molecular crosstalk between proliferating and differentiating cell networks to properly develop.^1^ During embryogenesis, perturbations in the growth environment as well as genetic aberrations can substantially impact this tenuous molecular milieu.^2^ While considerable progress has been made in studying isolated genetic mutations leading to syndromic and non-syndromic craniofacial disorders^3,4^, the molecular-genetic mechanisms driving morphogenetic events specific to craniofacial structures remain poorly understood. Such gaps in our understanding have restricted clinical treatment options for patients affected by common developmental anomalies, such as cleft lip and palate.^5^ Thus, there is a strong biologic rationale for a thorough investigation of basic molecular mechanisms driving craniofacial structure morphogenesis, which may pave the way toward translatable therapeutic developments for patients.

Among the critical structures of the craniofacial complex, the palate (an anatomic bridge separating oral and nasal cavities) is of particular interest – given its key functional impact in speech and swallowing.^6^ Palatogenesis is a highly regulated and tightly controlled process involving cascades of key genes and signaling pathways which are expressed in an orchestrated manner.^7^ For the palate to properly form in utero, several key developmental milestones must be achieved during the first trimester of gestation. Starting around the fourth week of human development, cranial neural crest cells (cNCCs) migrate from the dorsal edge of the rostral neural tube to form paired maxillary prominences as well as the frontonasal prominence surrounding the primate oral cavity.^8^ The nasal pits then take form, developing into paired medial and lateral nasal processes from the frontonasal prominence by the fifth week. The sixth week then brings about the merging of medial nasal processes with the maxillary processes to form the upper lip and primary palate; simultaneously, bilateral outgrowths from the maxillary processes, termed the palatal shelves, grow vertically along either side of the tongue. Mandibular growth in week seven further allows for descent of the tongue and the elevation of the palatal shelves to a more horizontal position. Proliferation and migration of the palatal shelf progenitor cell populations (epithelium and mesenchyme) leads to the formation of the midline epithelial seam – triggering the onset of the palatal fusion event. By the tenth week, the secondary palate fuses with the primary palate and nasal septum, allowing palatal mesenchyme to differentiate into bony and muscular elements. These fusion processes of primary and secondary palate components are complete by week 12, by which time the secondary palate is divided along the anterior-posterior axis into the bony (hard) palate and the muscular (soft) palate.

To better understand the molecular basis of these key developmental milestones during palatogenesis, genome-wide association studies (GWAS) have identified several transcription factors which influence risk to cleft palate.^9,10^ Among these identified transcription factors is PAX9 (paired homeobox domain-9), a master orchestrator of developmental patterning associated with proper morphogenesis of the axial skeleton, limbs, and craniofacial complex.^11^ Mechanistic studies have pointed to the importance of PAX9-dependent Wnt signaling during palatal development, including its connection to palatal osteogenesis.^12–14^ Meanwhile, PAX9’s role in the developmental events immediately preceding elevation and fusion of the palatal shelves has remained relatively underexplored. The aim of the present study was to test the hypothesis that PAX9 regulates the differentiation and patterning of progenitor cell populations prior to the osteogenic switch observed at the stage of palatal fusion. We hypothesized that PAX9’s upstream regulatory role as a patterning transcription factor extends beyond the development of palatal bone, including other mesenchyme-derived cell types and extracellular components.

To address this hypothesis and to better understand the key effector-ligand signaling interactions within the palatal shelves during embryogenesis, we performed the first high-resolution whole-transcriptome spatial RNA-sequencing study focused on the palate. Then, we further dissected the spatial transcriptomic expression profiles of target signaling networks via highly multiplex targeted single cell spatial profiling analyses (Xenium *in situ*, 10x Genomics, Inc.) using a custom designed panel of 350 unique genes specific to the developing palate. Finally, the key genetic targets identified from these high scale gene expression assays were validated via high-resolution multiplex RNAscope immunofluorescent in situ hybridization. Together, these analyses unveil a novel role of the transcription factor PAX9 in the embryonic palate.

## RESULTS AND DISCUSSION

### Whole Transcriptome Single Cell Spatial Sequencing in Cleft Palate Model Reveals Disruption in Wnt Signaling and Collagen Organization

**Figure 1.**
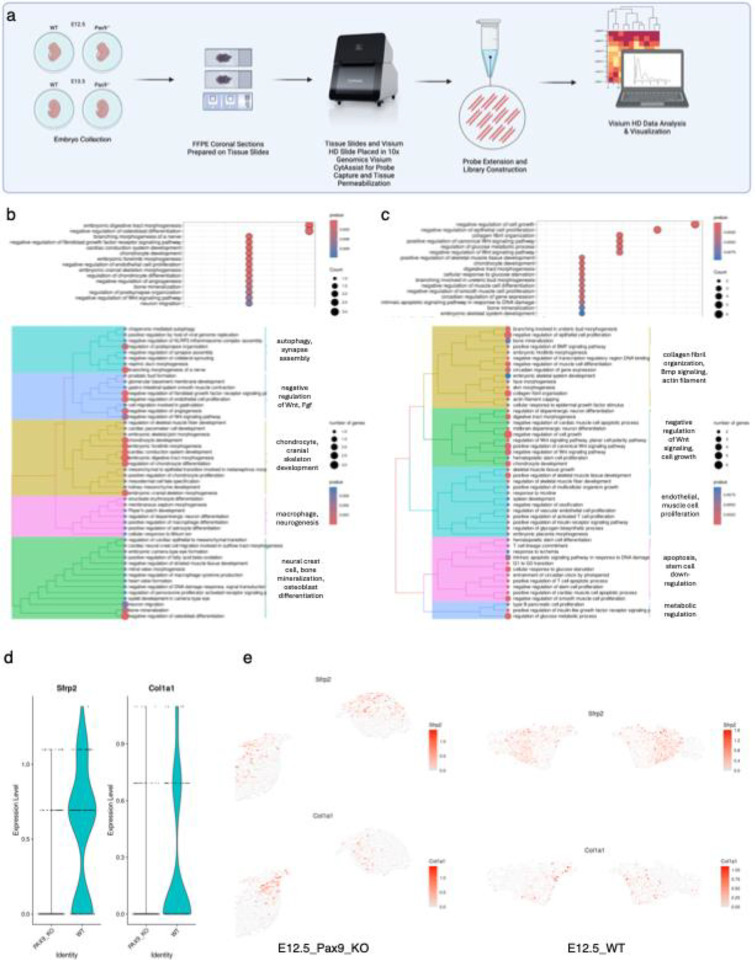
Whole Transcriptome Single Cell Spatial Sequencing in Cleft Palate Model Reveals Disruption in Wnt Signaling and Collagen Organization. a) Schematic representation of workflow for first whole transcriptome spatial sequencing in normal and abnormal palate development model. b-c) Functional enrichment analysis (FEA) showing an over representation test of the top 50 significant Biological Processes-Gene Ontology (BP-GO) terms of (b) E12.5 and (c) E13.5 wild type vs KO embryonic palate tissues from Visium HD whole transcriptome spatial analysis. d-e) Violin plots (d) and spatial distribution maps (e) of differentially expressed genes, *Sfrp2* (Wnt agonist) and *Col1a1* (critical extracellular matrix structural component) both in the presence (WT) and absence (KO) of the functional protein, PAX9.

To dissect the spatial gene expression landscape of the embryonic palate at its highest resolution to-date, we profiled embryonic day (E) 12.5 and E13.5 murine palate tissues in both normal (wild-type) and cleft (*Pax9*^−/−^) palate model organisms. We generated the first whole transcriptome spatial map of palate development at a single-cell level via Visium HD spatial RNA-seq (10x Genomics, Inc.). Embryos from both time points (n=2 embryos, n=4 palatal shelves per stage) were processed and sequenced together to avoid batch effect in differential analysis (Figure 1A).

To differentially analyze the molecular impact of the loss of the transcription factor, Pax9, we ran functional enrichment analysis (FEA) for both E12.5 (Figure 1B) and E13.5 (Figure 1C) showing an over representation test of the top 50 significant Biological Processes-Gene Ontology (BP-GO) terms. Upon differential comparison of the wild-type (WT) E12.5 to the *Pax9*^−/−^ palate of the same age, the top biological processes of significant change included: autophagy, synapse assembly; negative regulation of Wnt, Fgf; chondrocyte, cranial skeleton development; macrophage, neurogenesis; and neural crest cell, bone mineralization, and osteoblast differentiation (Figure 1B). In the E13.5 differential analysis, the loss of Pax9 in the palate exposed significant alterations in collagen fibril organization, Bmp signaling, actin filament assembly; negative regulation of Wnt signaling, cell growth; endothelial cell, muscle cell proliferation; apoptosis, stem cell down-regulation; and metabolic regulation (Figure 1C).

Spatial mapping of key genes associated with Wnt signaling regulation (*Sfrp2*) and collagen fibril organization (*Col1a1*) allowed for quantitative spatial comparison of expression patterns between WT and *Pax9*^−/−^ palatal shelves (Figures 1D and 1E). Each of these marker genes were identified to be downregulated along the most medial regions of the palatal shelves as early as E12.5 in development.

While the most sensitive phenotype in humans carrying heterozygous PAX9 variants is oligontia (congenital absence of six or more permanent teeth), a growing body of literature supports the functional interplay of PAX9 in the development of non-tooth oral structures of neural crest-derived mesenchyme.^15,16^ As the palatal shelves are similarly derived from cranial neural crest cells, the functional role of Pax9 in the setting of failed palatal mesenchymal patterning is likely preeminent.^17,18^

### Highly Multiplex Spatial Transcriptomic Analysis of Murine Embryonic Palate Using Novel Computational Pipeline

**Figure 2.**
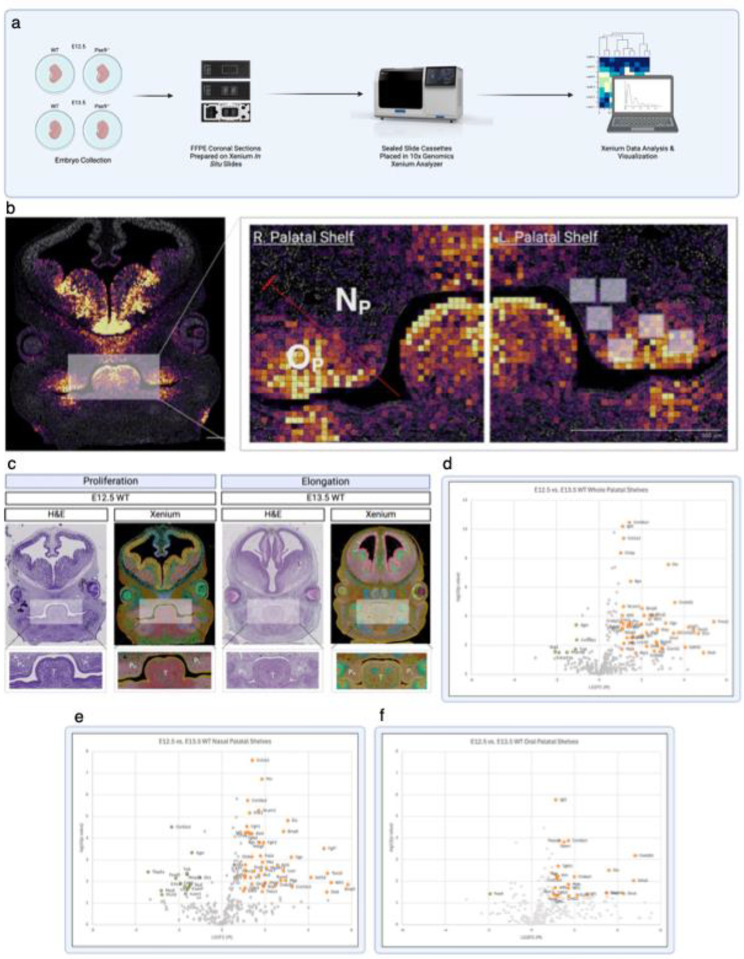
Highly Multiplex Spatial Transcriptomic Analysis of Murine Embryonic Palate Using Novel Computational Pipeline. a, c) Murine embryos were explanted on embryonic days 12.5 and 13.5 – key stages of palatogenesis – then formalin fixed, paraffin embedded prior to coronal sectioning for spatial transcriptomic pipelines. b) Overlaid co-expression of *Shh* and *Ptch1* clearly define sub-regions (oral versus nasal domains) of palatal shelves for refined spatial analyses using 10X Genomics Xenium *In Situ*. c) Morphological transition from proliferation to elongation characteristic of normal palatal development visualized with H&E staining and 10X Genomics Xenium Explorer v3.0 on E12.5 and E13.5. P_R_ right palatal shelf, P_L_ left palatal shelf, T tongue. d – f) Volcano plots of differential gene expression highlight statistically significant (P-value < 0.05, |LG_2_FC| > 1) upregulation (red) and downregulation (blue) of genes identified in transcriptomic comparison of the whole palatal shelves (d), nasal domains (e), and oral domains (f) from E12.5 and E13.5. All spatial transcriptomic analyses derived from n = 4 palatal shelf biological replicates. Not pictured: (d) Tnn (p-value = 0.008112, LG_2_FC = 8.369539); (e) Tnn (p-value = 0.003351, LG_2_GC = 8.942666); (f) Fmod (p-value = 0.00019, LG_2_FC = 7.12479); See also Figure S1.

To further analyze the expression patterns of these significant biological processes identified in our whole transcriptome single cell spatial analysis, we designed a highly multiplex spatial mRNA in situ assay for embryonic palate tissues using a custom genetic panel and a novel computational pipeline (Figures 2A, S4). Xenium In Situ (10x Genomics, Inc.) was employed on a separate batch of age matched and genotype matched samples from our spatial RNA-seq analysis described above. Sub-regional analysis of each palatal shelf included in this targeted validation of spatial sequencing via overlaid co-expression of *Shh* and *Ptch1* to highlight the oral palatal domain versus the nasal domain (Figure 2B) as described previously.^19^ First, the morphological transition from proliferation to elongation characteristic of normal palatal development was visualized with hematoxylin & eosin (H&E) staining and 10X Genomics Xenium Explorer v3.0 on WT embryos in the mid-coronal plane of E12.5 and E13.5. Based on our subregional comparison between developmental stages, volcano plots of differential gene expression were used to highlight statistically significant (P-value < 0.05, |LG_2_FC| > 1) upregulation (red) and downregulation (blue) of genes identified in transcriptomic comparison of the whole palatal shelves (48 upregulated, 6 downregulated) (Figure 2D), nasal domains (48 upregulated, 15 downregulated) (Figure 2E), and oral domains (26 upregulated, 1 downregulated) (Figure 2F) from E12.5 and E13.5. This spatial transcriptomic roadmap of palatal development from E12.5 to E13.5 allowed for the verification of normal genetic expression trajectories, specifically with the up-regulated expression of extracellular matrix component genes (e.g., *Col1a1*, *Eln*, *Bgn*, etc.) as well as patterning molecules (e.g., *Prrx1*, *Alx1*, *Dlx1*, etc.), allowing us to compare this trajectory with that of the *Pax9*^−/−^ palatal shelves (see Figure S1).

### The *Pax9*^−/−^ Cleft Palate Demonstrates Disrupted Spatiotemporal Genetic Programs Across Oral and Nasal Domains

Next, we differentially analyzed the spatial transcriptomic profiles of abnormal (cleft, *Pax9*^−/−^) across both E12.5 and E13.5 developmental stages via sub-regional highly multiplex mRNA localization (Figure S2A). Aberrancies in the morphological transition from proliferation to elongation in the *Pax9*^−/−^ cleft palate at both E12.5 and E13.5 were visualized via H&E and Xenium Explorer, highlighting the lack of proper extension of the mesial palatal shelves bilaterally compared to the WT palate of the same age. Volcano plots of differential gene expression highlight statistically significant (P-value < 0.05, |LG2FC| > 1) upregulation (red) and downregulation (blue) of genes identified in transcriptomic comparison of the whole palatal shelves (48 upregulated, 8 downregulated) (Figure S2B), nasal domains (35 upregulated, 9 downregulated) (Figure S2C), and oral domains (60 upregulated, 5 downregulated) (Figure S2D) from E12.5 to E13.5.

### Palatal Shelves in *Pax9*^−/−^ Model Characterized by Aberrant Expression of Homeobox Genes, Wnt Modulators & Effectors, and ECM Proteins

**Figure 3.**
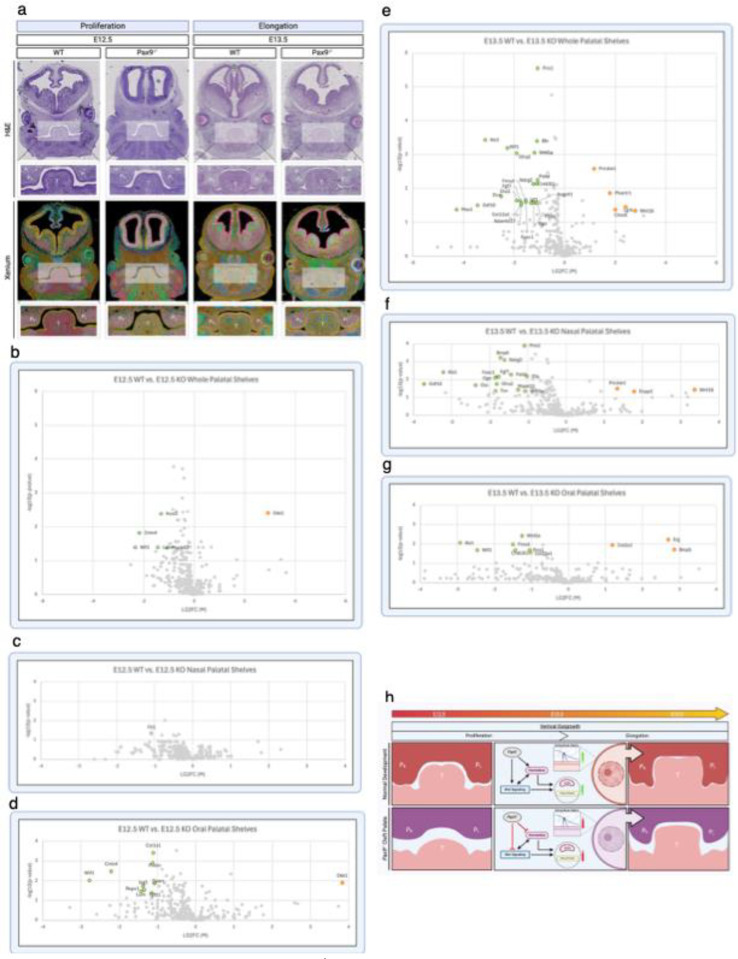
Palatal Shelves in *Pax9*^−/−^ Model Characterized by Aberrant Expression of Homeobox Genes, Wnt Modulators & Effectors, and ECM Proteins. a) Side-by-side visual comparison using H&E staining and 10x Genomics Xenium Explorer v3.0 highlights morphological differences in palatal development from E12.5 to E13.5 in wildtype versus *Pax9*^*−/−*^ models. P_R_ right palatal shelf, P_L_ left palatal shelf, T tongue. b – d) Volcano plots of differential gene expression highlight statistically significant (P-value < 0.05, |LG2FC| > 1) upregulation (red) and downregulation (blue) of genes identified in transcriptomic comparison of the whole palatal shelves (b), nasal domains (c), and oral domains (d) in E12.5 wildtype versus *Pax9*^−/−^ models. All spatial transcriptomic analyses derived from n = 4 palatal shelf biological replicates per stage of development. e – g) Volcano plots representing the same sequence of transcriptomic comparisons at E13.5. h) Schematic representation of identified genetic programs altered in the absence of Pax9 during palatal development. All spatial transcriptomic analyses derived from n = 4 palatal shelf biological replicates per stage of development.

Unsurprisingly, where we identified the greatest transcriptomic shift upon differential expression analysis was the comparison between WT and *Pax9*^−/−^ palatal shelves of the same stage in development (Figure 3A). Our analysis identified differential gene expression in four key gene categories: homeobox genes (*Alx1, Prrx1, Msx1*, and *Gsc*); Wnt signaling modulators (*Dkk1, Rspo1, Lgr5, Wif1, Sparc, Prickle1, Tnn*, and *Jag1*); Wnt signaling effectors (*Wnt5a* and *Wnt16*); and extracellular matrix proteins (*Lum, Ogn, Fmod, Dcn, Eln, Col1a1, Col12a1, Col2a1, Col23a1, Postn, Adamts17*, and *Palld*).

Differential analysis comparing transcription in the whole palatal shelves at E12.5 revealed six differentially expressed genes (Figure 3B). Of the six differentially expressed genes, one (*Dkk1*) was upregulated and five (*Mapk13*, *Wif1*, *Lum*, *Cntn4*, and *Nos3*) were downregulated in the *Pax9*^−/−^ embryos relative to WT controls. The nasal palatal domain subregional analysis revealed only one differentially expressed gene, *Flt1*, to be downregulated in the *Pax9*^−/−^ model relative to WT control (Figure 3C). The nasal palatal domain subregional analysis at E12.5 revealed 10 differentially expressed genes; one (*Dkk1*) was upregulated and nine (*Tgfb1*, *Lum, Rspo1, Jag1, Sparc, Wif1, Cntn4, Postn*, *Col1a1*) were downregulated in *Pax9*^−/−^ cleft (Figure 3D).

At E13.5, our differential analysis revealed 27 differentially expressed genes within the whole palatal shelves of *Pax9*^−/−^ embryos compared to WT controls (Figure 3E). Of the 27 differentially expressed genes, five (*Prickle*, *Phactr1, Chodl, Lgr5*, and *Wnt16*) were upregulated and 25 (*Msx1, Lgr5, Gdf10, Col12a1, Adamts17, Igf1, Pdpn, Ogn, Angptl1, Fmod, Fgf7, Dio3, Foxc1, Dcn, Creb3l1, Ndrg2, Palld, Prickle1, Gfra2, Wnt5a, Wif1, Eln, Alx1*, and *Prrx1*) were downregulated. In the nasal subregion at E13.5, 18 differentially expressed genes were identified; three (*Prickle1*, *Enpp2*, and *Wnt16*) were upregulated and 15 (*Wnt5a, Tnn, Mapk13, Gsc, Gdf10, Gfra2, Ogn, Foxc1, Fgf7, Eln, Palld, Alx1, Ndrg, Bmp6*, and *Prrx1*) were downregulated (Figure 3F). In the oral sub-domain, 10 differentially expressed genes were identified, three of which were upregulated (*Col2a1*, *Erg*, and *Bmp5*) while 7 (*Col23a1, Wif1, Prrx1, Creb3l1, Fmod, Alx1, Wnt5a*) were downregulated in the *Pax9*^−/−^ cleft palate (Figure 3G). Taken together, this targeted validation of spatial RNA expression profiles between normal and cleft palate samples identified significant alterations to homeobox gene expression, Wnt signaling modulation, and extracellular matrix structural components.

Canonical Wnt/β-catenin signaling has been implicated downstream of Pax9 in the craniofacial complex, perhaps best exemplified by pivotal work demonstrating reduced Wnt activity in palatal shelves in the absence of functional Pax9.^12–14^ This pathway has been shown to be crucial for intramembranous bone development, which may explain the reversal of cleft palate phenotype with genetic and pharmacological modulation of Wnt signaling in the setting of *Pax9* deficiency.^13,14^ However, these studies also helped delineate that Pax9 is involved in other overlapping pathways and processes in addition to Wnt signaling, as observed by the prevailing phenotype of tooth agenesis despite genetic and pharmacologic intervention.^20^ Thus, the identification of homeobox gene and ECM network aberrations in the present study provide additional insights to the expansive role of Pax9 in the developing palate.

### Xenium In Situ RNA Localization of Differentially Expressed Genetic Markers in *Pax9*^−/−^ Cleft Palate

**Figure 4.**
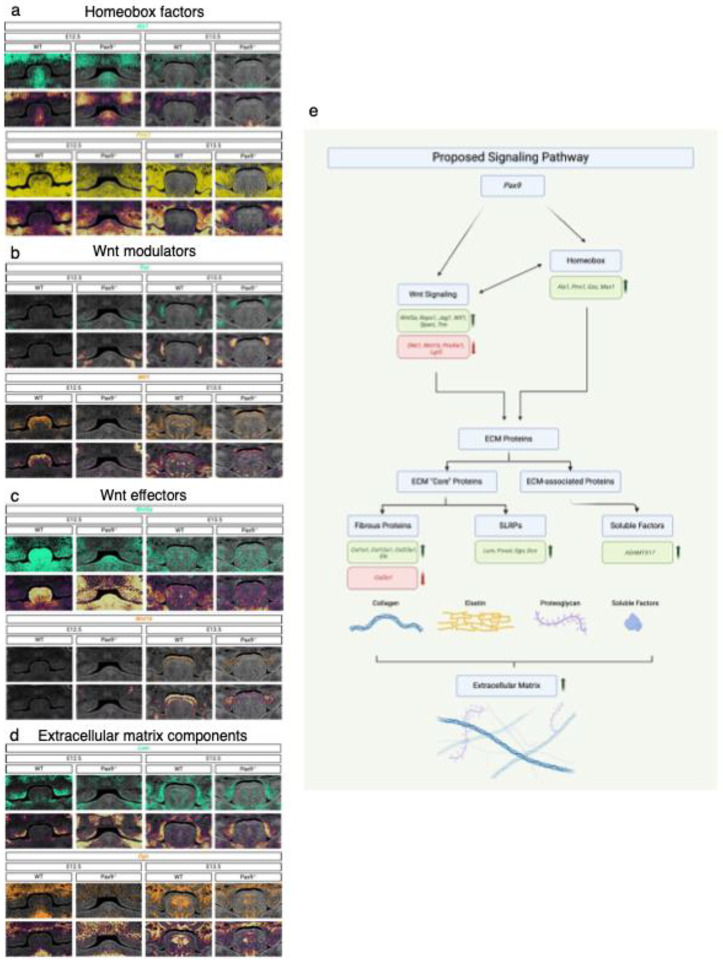
Xenium In Situ RNA Localization of Differentially Expressed Genetic Pathways in *Pax9*^−/−^ Cleft Palate. a – d) Spatial resolution of homeobox (a), *Wnt* modulator and effector (b, c), and ECM protein (d, e) gene transcripts (above) and corresponding density maps (below) validates prior differential gene expression analysis suggesting spatiotemporal disruption of genetic pathways in E12.5 and E13.5 *Pax9*^−/−^palatal shelves; (e) Schematic summary of proposed signaling disruption in the absence of Pax9 in embryonic palatal shelves, contributing to the cleft palate phenotype observed.

To better understand the spatial profiles of the quantified transcriptomic findings in this novel computational pipeline, we generated spatial plots and density maps of gene expression to visualize transcript localization within the palatal shelves in both WT and *Pax9*^−/−^ embryos at E12.5 and E13.5. Spatial maps of homeobox genes (*Alx1* and *Prrx1*) (Figure 4A), Wnt modulator genes (*Tnn* and *Wif1*) (Figure 4B), Wnt effector genes (*Wnt5a* and *Wnt16*) (Figure 4C), as well as ECM component small leucine-rich proteoglycans (SLRPs) (*Lum* and *Ogn*) (Figure 4D), each delineate aberrant expression patterns in the absence of Pax9 in the embryonic palate.

Together, these overlapping signaling networks’ aberrant spatial gene expression patterns in the *Pax9*^−/−^ palate likely point to the complex interplay and requirement for crosstalk between patterning transcription factors (such as those homeobox proteins identified herein) and Wnt signaling molecules to produce a viable ECM framework. Given the fully penetrant phenotype of cleft secondary palate observed in the *Pax9*^−/−^ model, this crosstalk may be required to undergo requisite vertical elongation and eventual midline growth to achieve palatal fusion. While the genetic data demonstrated in the present study hint at this potential mechanism (Figure 4E), further functional studies will be prudent to verify this intrinsic interaction between Pax9, Wnts, and the palatal ECM framework during embryogenesis.

### Validation of High Throughput Spatial Transcriptomic Xenium In Situ Gene Expression via RNAscope Multiplex

As this study represents one of the very first studies to apply the Xenium In Situ spatial RNA localization technology to-date, it was important for us to further validate our key transcriptomic targets by way of an established RNA profiling platform, as we did in our recent work.^12,21^ To that end, we performed a targeted multiplex RNAscope immunofluorescence in situ hybridization to validate in sub-cellular resolution the expression profiles of key genes in our differential analysis for both developmental stages and genotypes (Figures S3A–C). This included the homeobox genes (*Alx1, Prrx1*), Wnt modulators and effector genes (*Rspo1, Wif1, Sparc, Wnt5a*), fibrous ECM protein genes (*Eln, Col1a1*) and the SLRP family gene, *Bgn*. All these genes, which were first identified from whole transcriptome and highly multiplex in situ analyses, were validated via RNAscope with concordant expression patterns in all palatal shelves analyzed.

As observed in multiple platforms of spatial transcriptomic analysis, Pax9 may be involved in mesenchymal patterning and organization, with likely contribution to Wnt signaling and ECM structural organization in the developing palate (Figure 4F).^22^ As a patterning transcription factor involved early in development, Pax9’s role in recruiting proliferating and differentiating neural crest-derived mesenchyme to form intramembranous bone and its surrounding ECM architecture likely contributes to the observed cleft phenotype in its absence.^23^

By employing these next-generation spatial sequencing platforms in the embryonic palate, we hope to empower the craniofacial biology research community with abundant access to these novel datasets. With these tools and the technologies available for downstream analysis, we hope to further identify developmental signaling pathways which go awry in the setting of cleft palate disorders. Together, the results from this study compiling novel statistical and multimodal spatiotemporal transcriptomic analysis approaches reveal a central role for the transcription factor, Pax9, in proper patterning and organization of ECM in the embryonic palate. As a key step toward laying the framework for identifying key molecular targets these data can be used for translational studies aimed at developing effective therapies for human palatal clefts.

## METHODS

### Animals

All animal procedures were approved by the National Institutes of Health, National Institute of Child Health and Human Development Animal Care and Use Committee (ACUC), under Animal Study Protocol (ASP) #21–031. C57BL/6J mice were obtained from the Jackson Laboratory. Inbred strain of female C57BL/6 mice were utilized for all experiments. *Pax9*^+/−^ mice were provided by Dr. Rulang Jiang (Cincinnati Children’s Hospital) and generated as described previously (Zhou et al. 2013). Timed pregnancies were conducted via vaginal plug identification, with day 0.5 indicating date of identification. To assure accurate developmental stage comparison, embryos comparatively analyzed were of the same litter, with the same C57BL/6 background. Genotyping of all mice included in this study was performed by Transnetyx, Inc, as well as sex determination for inclusion of both male and female sex (at least one biological male and one biological female per comparison group). This study conforms to the ARRIVE 2.0 guidelines.

### Visium HD Spatial RNA-sequencing

All steps from 10X Genomics’ FFPE Visium HD Spatial workflow were followed. In brief, high-definition spatial RNA sequencing (Visium HD) slides are coated with an array of poly-T primers, which encode unique spatial barcodes. These barcodes contain thousands of encoded oligonucleotides within a catchment frame of 6.5 × 6.5 mm. The oligonucleotides are selectively hybridized with the 3’ end of mRNA eluted upon tissue permeabilization, enabling true single-cell-seq-like mRNA sequencing when binned to 2×2 micron resolution. FFPE tissue was sectioned directly onto the barcoded slide, H&E stained, and then underwent enzymatic permeabilization, which allowed for mRNA release and subsequent capture by primer-coated slides. These mRNA molecules are visualized through the incorporation of fluorescent nucleotides into the complementary DNA (cDNA) synthesis process. The resolution of unique spatial barcoding in situ allows the matching of RNA abundance with the original spatial location in the tissue section, providing a whole-transcriptome RNA-sequencing at single-cell resolution with 2-dimensional spatial relation.

### Xenium In Situ mRNA Localization and Analysis

Xenium In Situ sub-cellular mRNA detection technology was employed as previously reported following all manufacturer guidelines and specifications.^24^ We custom designed a 350-plex targeted gene panel as previously reported^12^ to detect mRNA expression for cell type identification as well as signaling pathway interactions selected and curated primarily based on single cell atlas data we generated in our previous work on palate development.^21^ For this assay, we formalin fixed and paraffin embedded 2 biological replicates (n=4 palatal shelves) of *Pax9*^−/−^ as well as *Pax9*^+/+^ mouse embryos at embryonic days 12.5 and 13.5. Whole heads were processed, embedded, and sectioned (4 um) to the level of the 1^st^ molar tooth bud. All 4 samples were assayed in a single batch under identical conditions. The post-Xenium H&E staining followed Demonstrated Protocol CG000160 from 10x Genomics, Inc.

### Computational Analysis of Visium HD Data

We created custom bins of 2×2 μm Visium HD spatial gene expression data based on the identified cell nuclei in the microscope image of the tissue used in the Visium HD assay. The cell nuclei were automatically segmented using the StarDist AI model, and improperly segmented nuclei were filtered by assessing nuclei size and unique molecular identifier (UMI) counts. We directly removed mitochondrial (mt)RNA reads to avoid data skewing, simplify analysis, and focus on specific nuclear RNA transcript populations as previously exemplified.^25,26^ Gene expression was quantified at single-cell resolution by mapping spatial transcriptomic data to the segmented nuclei. Downstream analysis was conducted using Seurat V5 in R, including normalization and scaling based on UMI counts via the SCTransform (v2) algorithm. We identified highly variable genes across the segmented single cells, performed principal component analysis (PCA), uniform manifold approximation and projection (UMAP), and unsupervised clustering, and identified differentially expressed cell-type-specific markers. Specifically, the differentially expressed markers were identified using the “roc” method within Seurat V5, which evaluates each gene’s ability to distinguish between two cell groups using receiver operating characteristic (ROC) curve analysis, returning a ranked matrix of predictive power based on area under the curve (AUC) values.

Differentially expressed genes (DEGs) in E12.5 WT vs. E13.5 WT, E12.5 WT vs. E12.5 KO, and E13.5 WT vs. E13.5 KO were identified using Model-based Analysis of Singlecell Transcriptomics (MAST) in R, with the criteria of |LG_2_FC| > 1 and false discovery rate (FDR) < 0.01. The identified DEGs were subsequently used for over-representation analysis (ORA) to identify enriched biological functions through the Gene Ontology (GO) and Kyoto Encyclopedia of Genes and Genomes (KEGG) databases using the clusterProfiler package, with criteria of P-value < 0.05 and gene counts > 1.

### Computational Analysis of Xenium In Situ Data

At the time of this analysis, no publicly available open-access software would allow us to perform the statistical tests necessary to achieve our project aims. Thus, all analyses were generated using a custom designed data pipeline in Microsoft Excel.

Our analysis began in the 10x Genomics open-access software, Xenium Explorer v3.0. First, we sought out to develop a method for reliably dividing the embryonic palatal shelves into nasal palatal (NP) and oral palatal (OP) regions for more precise region-specific analysis. We were able to define a reproduceable oronasal axis in both our WT and *Pax9*^−/−^mouse models by isolating the co-expression of *Shh* and *Ptch1* on the Xenium Explorer v3.0 density map. During embryonic development, *Shh* expression is restricted to the epithelium of the oral side of the palatal shelves^27^, while known target gene, *Ptch1*, is localized to the nearby mesenchyme.^28^ Thus, by isolating the co-expression of *Shh* and *Ptch1*, we were able to successfully distinguish the oral region of the palatal shelves from the nasal region.

Next, we defined our regions of interest (ROIs). ROIs were standardized by surface area (6400 um^2^ +/− 100 um). Three ROIs were selected from each side of the oronasal axis, per palatal shelf; thus, a total of 12 ROIs were generated per section. The ROIs were exported as .csv files and opened using Microsoft Excel. Raw data sets were produced using the transcript density data from these files.

To perform differential gene expression (DGE) analysis, we calculated both p-values and Log2 fold-change (LG_2_FCs) from our transcript density data sets. While the p-value is a measure of the statistical significance of the expression difference between comparison groups, the LG_2_FC is an estimate of the log_2_ ratio of the change in expression.^12^ Genes that displayed a p-value < 0.05 and LG_2_FC > 1 were considered differentially expressed for each of our comparison groups.

LG_2_FCs were calculated using raw data. By contrast, to obtain p-values, we first normalized our data using a LG_2_(x + 1) transformation to reduce variance. We then tested for equal variance using an F-test (F-critical = 0.05). Based on the F-statistic, a student’s (equal variance) or Welch’s (unequal variance) T-test was deployed. The Benjamini-Hochberg procedure was performed to obtain p-values adjusted for false discovery rates. Finally, the LG_2_FC and −Log_10_(p-value) were mapped onto volcano plots to visually represent the differentially expressed genes.

## Resource availability

### Lead contact

Dr. Jeremie Oliver Piña (jeremie.pina@nih.gov)

### Materials availability

All materials used for this study have been reported in the Methods section; any further questions regarding materials can be directed to the first author of correspondence, Dr. Jeremie Oliver Piña (jeremie.pina@nih.gov).

### Data and code availability

All results and analysis data are available in the main text or the supplementary file. Additional information and materials are available from the corresponding author upon reasonable request. All raw data from spatial RNA-sequencing is accessible open-access at the accession: GSE284271. The codes for analysis of 10x Visium HD Spatial RNA-sequencing were deposited into the GitHub at https://github.com/LabOnoM/GSE284271.

## Supplementary Material

1

## Figures and Tables

**Table:** Key Resources Table

REAGENT or RESOURCE	SOURCE	IDENTIFIER
Antibodies		
Opal 520 Reagent Pack	Akoya Biosciences	FP1487001KT
Opal 570 Reagent Pack	Akoya Biosciences	FP1488001KT
Opal 690 Reagent Pack	Akoya Biosciences	FP1497001KT
Opal Polaris 780 Reagent Pack	Akoya Biosciences	FP1501001KT
Chemicals, peptides, and recombinant proteins		
ProLong Gold Antifade Mountant	Thermo Fisher Scientific	P36930
PBS	Sigma	Cat# D8537
DAPI	Invitrogen	Cat# D1306
RNAscope Wash Buffer	ACDBio/Bio-Techne	Cat# 310091
Critical commercial assays		
Visium HD Spatial RNA-sequencing	10X Genomics, Inc.	Cat# 1000676
Xenium In Situ RNA Localization	10X Genomics, Inc.	Cat# 1000672
RNAscope Multiplex Fluorescent Reagent Kit v2	ACDBio/Bio-Techne	Cat# 323100
RNAscope 4-Plex Ancillary Kit	ACDBio/Bio-Techne	Cat#323120
Deposited data		
Spatial RNA-sequencing raw data	Gene Expression Omnibus (GEO)	GSE284271
Experimental models: Organisms/strains		
Mouse: C57BL/6J	Charles River	JAX: 000664; RRID: IMSR JAX 000664
Mouse: *Pax9*^*null*^	Zhou et al. 2011^24^	N/A
Software and algorithms		
Xenium Explorer	10X Genomics, Inc.	Github: https://github.com/quentinblampey/spatialdata_xenium_explorer
Seurat	Satija Lab	Github: https://aithub.com/satiialab/seurat
Spatial Gene Expression Analysis Pipeline	Ziyi Wang	https://labonom.github.io/GSE284271/00.NIH_10xVisiumHD.html

## HIGHLIGHTS

- The first single-cell spatial RNA-sequencing study to date in a cleft palate model
- Aberrant ECM transcriptional programming networks identified in *Pax9*^−/−^ embryos
- Wnt signaling disruption within palatal mesenchyme spatiotemporally linked to ECM
- Wnt-driven ECM-targeting therapeutic strategies should be explored for palatal clefts
