# Supplementary material for: Single Cell Spatial Transcriptomics of the Murine Embryonic Palate Links Pax9 to Patterning and Organization of Extracellular Matrix Components
Source: Res Sq. 2025 Feb 19:rs.3.rs-5969552. Preprint. [Version 1] doi: 10.21203/rs.3.rs-5969552/v1 (PMC11875297; doi:10.21203/rs.3.rs-5969552/v1)
Supplement: 1 [file NIHPPrs5969552v1-supplement-1.pdf]

SUPPLEMENTAL FILE

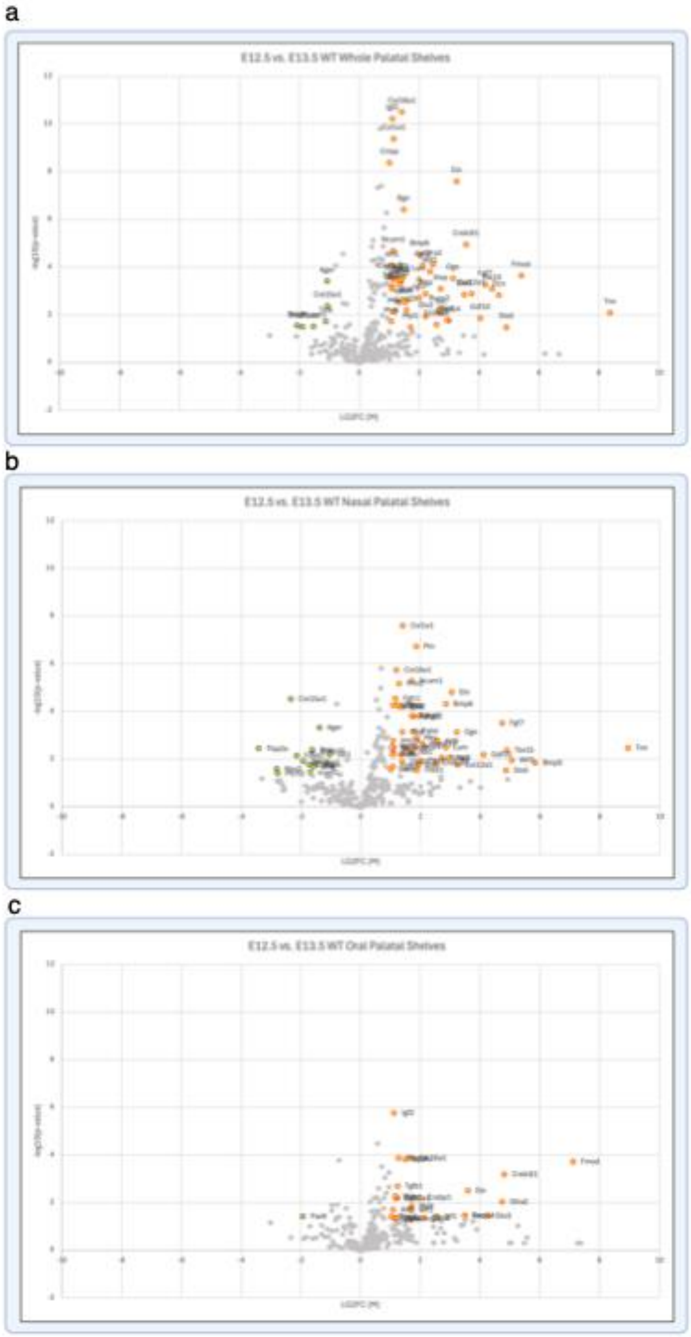

Figure S1. Xenium In Situ Spatial Analysis Clarifies Genetic Programs Underlying Palatogenesis from E12.5 to E13.5 in Normal Development.
a – c) Full size volcano plots of differential gene expression highlight statistically significant (P-value < 0.05, $|LG_2FC| > 1$) upregulation (red) and downregulation (blue) of genes identified in transcriptomic comparison of the whole palatal shelves (a), nasal domains (b), and oral domains (c) from E12.5 and E13.5, including the genes not pictured in Figure 2. All spatial transcriptomic analyses derived from n = 4 palatal shelf biological replicates.

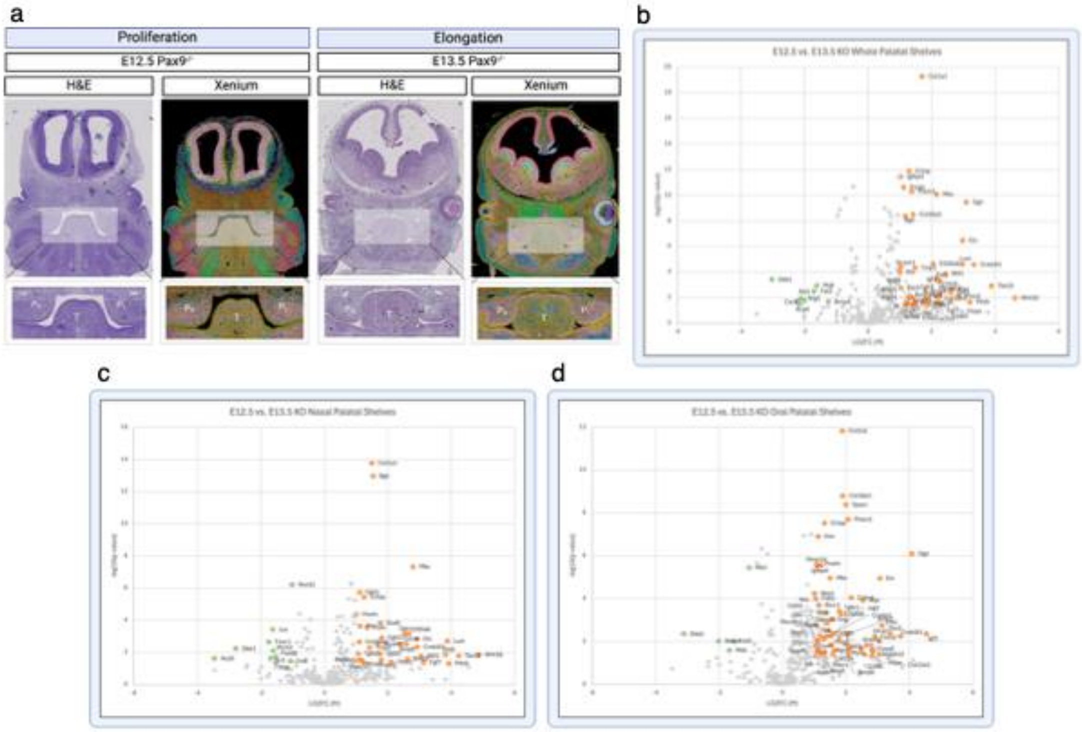

Figure S2. The *Pax9*$^{-/-}$ Cleft Palate Demonstrates Disrupted Spatiotemporal Genetic Programs Across Oral and Nasal Domains.
a) Aberrancies in the morphological transition from proliferation to elongation in Pax9$^{-/-}$ cleft palate visualized with H&E staining and 10X Genomics Xenium Explorer v3.0 on E12.5 and E13.5. P_R right palatal shelf, P_L left palatal shelf, T tongue. b – d) Volcano plots of differential gene expression highlight statistically significant (P-value < 0.05, |LG2FC| > 1) upregulation (red) and downregulation (blue) of genes identified in transcriptomic comparison of the whole palatal shelves (B), nasal domains (C), and oral domains (D) from E12.5 to E13.5. All spatial transcriptomic analyses derived from n = 4 palatal shelf biological replicates.

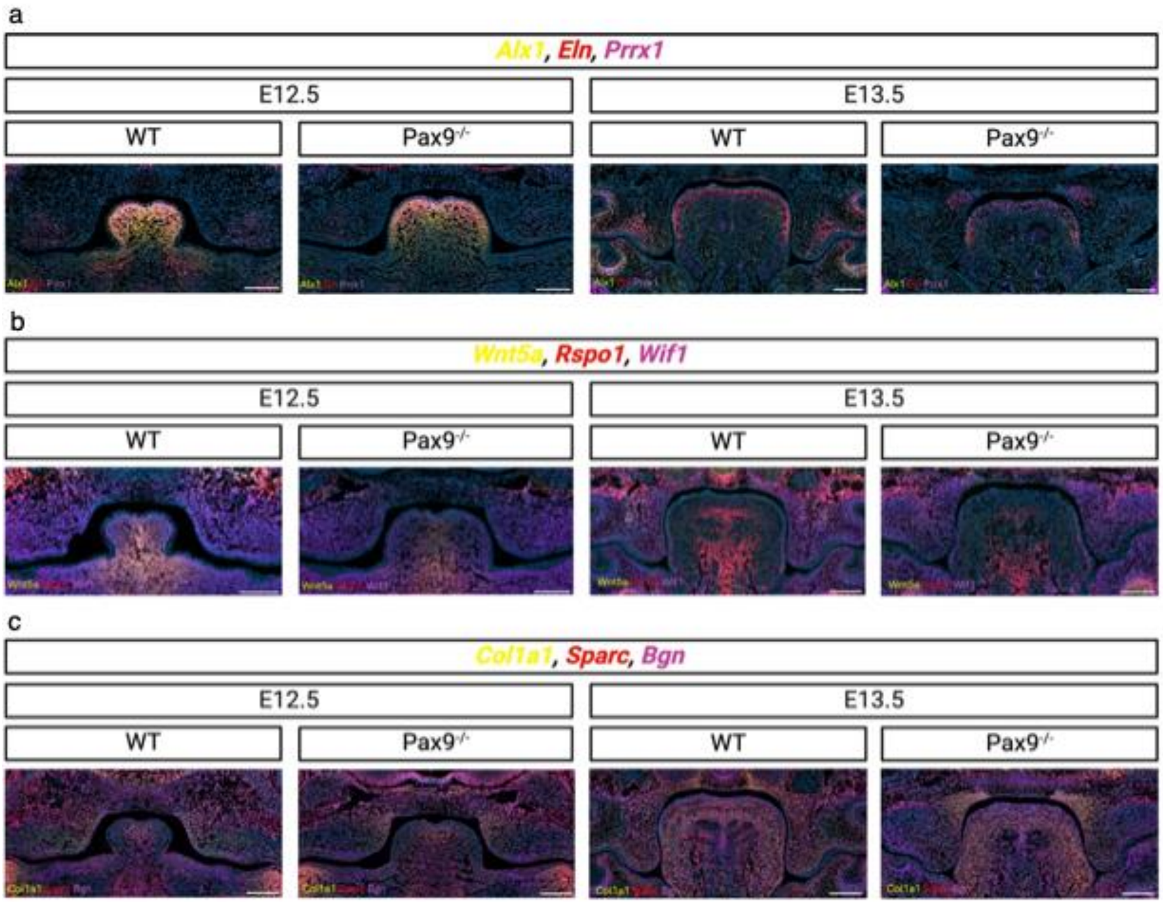

Figure S3. RNAscope Multiplex Validates Quantitative Spatial Transcriptomic Xenium In Situ Spatial Gene Expression Analysis
a – c) Homeobox genes, *Wnt* modulators and effectors, and genes encoding ECM proteins hybridized in situ to confirm spatial patterns of differentiation: *Alx1* and *Prrx1* (homeobox); *Rspo1*, *Wif1*, and *Sparc* (Wnt modulator); *Wnt5a* (Wnt effector); *Eln* and *Col1a1*(fibrous protein); *Bgn* (SLRP) in palatal shelves of E12.5 and E13.5 wildtype versus Pax9$^{-/-}$ models. All spatial transcriptomic analyses derived from n = 4 palatal shelf biological replicates per stage of development.

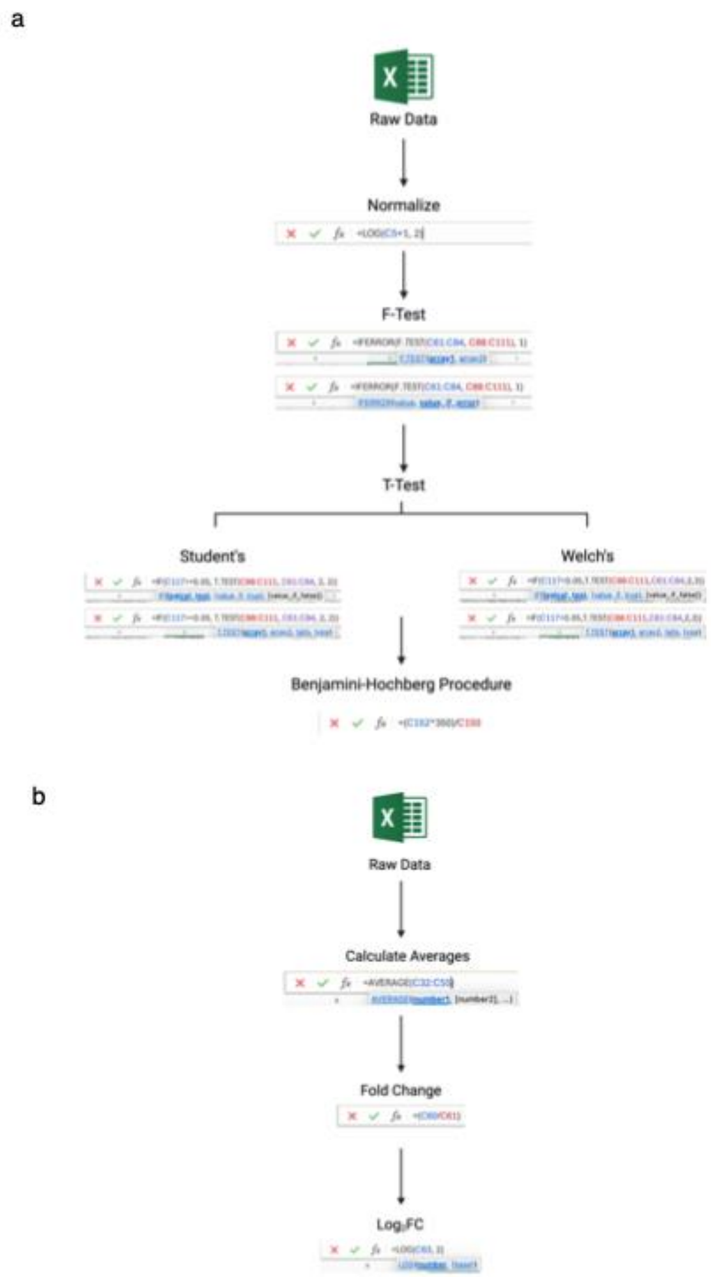

Figure S4. Statistical Pipelines for Xenium *In Situ* Differential Gene Expression Analysis
a) Diagram of the statistical pipeline for calculation of p-values from Xenium *In Situ* transcript density outputs. First, raw data outputs were $Log_2(X+1)$ transformed. Next, variance was calculated using an F-test ($\alpha = 0.0$). Equal variance was assumed in cases #DIV/0 error. For genes with equal variance (F-test p-value >=0.05), a Student's T-Test was performed. Alternatively, a Welch's T-Test was performed if variance was determined to be unequal (F-test p-value <0.05). Finally, the Benjamini-Hochberg procedure was deployed as a false discovery rate control. b) Diagram of the statistical pipeline for calculation of LG_2FCs from Xenium *In Situ* transcript density outputs. First, raw data was used to calculate the average transcript density data for each comparison group. Then, the fold change was calculated using the function shown and transformed via Log_2 transformation.
